# Magnitude, precision, and realism of depth perception in stereoscopic vision

**DOI:** 10.1186/s41235-017-0062-7

**Published:** 2017-05-24

**Authors:** Paul B. Hibbard, Alice E. Haines, Rebecca L. Hornsey

**Affiliations:** Department of Psychology, University of Essex, Wivenhoe Park, Colchester, Essex CO4 3SQ UK

**Keywords:** Depth perception, Binocular vision, Image quality, Stereopsis, Stereoscopic 3D, Binocular fusion

## Abstract

Our perception of depth is substantially enhanced by the fact that we have binocular vision. This provides us with more precise and accurate estimates of depth and an improved qualitative appreciation of the three-dimensional (3D) shapes and positions of objects. We assessed the link between these quantitative and qualitative aspects of 3D vision. Specifically, we wished to determine whether the realism of apparent depth from binocular cues is associated with the magnitude or precision of perceived depth and the degree of binocular fusion. We presented participants with stereograms containing randomly positioned circles and measured how the magnitude, realism, and precision of depth perception varied with the size of the disparities presented. We found that as the size of the disparity increased, the magnitude of perceived depth increased, while the precision with which observers could make depth discrimination judgments decreased. Beyond an initial increase, depth realism decreased with increasing disparity magnitude. This decrease occurred well below the disparity limit required to ensure comfortable viewing.

## Significance

The quality of the viewing experience for 3D displays depends on multiple factors, including the accuracy of perceived depth, the viewing comfort, and the realness and naturalness of the 3D experience. Depth realism is distinct from the amount of depth perceived and is important in creating a rewarding stereoscopic experience and a sense of presence and immersion. We assessed how stimulus parameters in a 3D display might be set to optimize the realism of stereoscopic depth. Specifically, we assessed the contribution of the magnitude and precision of perceived depth, and the fusion of the images into a single coherent percept, to depth realism. We found that increasing the depth range tended to increase depth realism only over a very small range of disparities, well below the level required to avoid visual discomfort. Our results suggest that relatively small amounts of binocular parallax are required to enhance the experience of realistic depth.

## Background

Binocular vision provides an important source of depth information that contributes to the performance of many everyday tasks (Bradshaw et al., 2004; Hayhoe, Gillam, Chajka, & Vecellio, 2009; Hibbard & Bradshaw, 2003; Keefe, Hibbard, & Watt, 2011; Loftus, Servos, Goodale, Mendarozqueta, & Mon-Williams, 2004; McIntire, Havig, & Geiselman, 2014; Melmoth & Grant, 2006; Patla, Niechwiej, Racco, & Goodale, 2002; Read, Begum, McDonald, & Trowbridge, 2013; Servos, Goodale, & Jakobson, 1992; Watt & Bradshaw, 2003). Binocular cues are valuable because they are very precise (e.g. Harris, 2004; McKee, 1983; Stevenson, Cormack, & Schor, 1989) and also because, unlike many other depth cues, they provide scaled metric depth information. This means that they allow the actual shape, size, and location of objects to be estimated.

The improvement of depth perception provided by stereoscopic cues is important in enhancing the user experience in stereoscopic three-dimensional displays (S3D). In a review of the enhancement in performance provided in S3D compared with non-stereoscopic displays, McIntire, Havig, and Geiselman (2012) concluded that stereoscopic displays lead to a clear improvement in performance on tasks requiring spatial understanding or the manipulation of objects and are somewhat useful for tasks requiring the judgment of the position or distance of objects and in finding, identifying, or classifying objects. In medical applications, stereoscopic displays have been shown to be particularly useful in improving visualization and diagnosis in medical imaging, and spatial orientation and performance in minimally invasive surgery (Held & Hui, 2011). In each of these cases, the improvements in performance are consistent with the enhanced information about 3D structure provided by stereoscopic cues.

Stereoscopic displays enhance the perceptual experience of depth, as well as improving performance on tasks requiring the use of depth information (McIntire et al., 2012). Ideally, an optimal display would create a 3D experience that was indistinguishable from a direct view of the real world (Banks, Hoffman, Kim, & Wetzstein, 2016). The creation of a realistic 3D experience is particularly important in creating a sense of presence in movies, games, and virtual reality (Freeman & Avons, 2000). The aim of the current study is to determine which aspects of the representation of stereoscopic depth are important in creating a convincing and realistic 3D experience.

Banks, Hoffman, Kim, and Wetzstein (2016) recently reviewed the hardware factors that affect the degree of realism experienced in 3D displays. Stimulus parameters that influence the realism of the 3D experience have also been assessed. These include image quality (Lambooij, IJsselsteijn, Bouwhuis, & Heynderickx, 2011; Seuntiens, Meesters, & IJsselsteijn, 2006), the separation between the left and right cameras and the focal length used in capturing the images and therefore the range of binocular disparities and how these relate to other depth cues (IJsselsteijn, de Ridder, & Hamberg, 1998). When complex images such as natural photographs are used for stimuli, the manipulation of binocular disparity tends to create a conflict between the depth specified by binocular disparity and that specified by pictorial cues. This is because, while the depth specified by disparity increases when the camera separation is increased, depth specified by pictorial cues remains unchanged. This conflict could potentially reduce the realism of the 3D experience. This conflict can be reduced by using stimuli in which the informativeness of non-stereoscopic depth cues is minimized. In the current study, we studied the effect of manipulating the disparity content of images on the apparent depth realism, using simple stimuli that allowed us to easily quantify the effect of binocular cues. We used this approach to allow us to focus on those aspects of the representation of stereoscopic depth that can be predicted, on theoretical grounds, to contribute to the realism of the experience.

The first factor under consideration is the magnitude of apparent depth. The relative disparity between two objects increases monotonically as their physical separation in the depth direction increases. If depth was perceived accurately, then it should increase in just the same way with the disparity present in the image. For relatively small disparities, this is what is observed (Ogle, 1952). While depth is still seen for larger values, there is no further increase in the amount of depth seen with the size of the disparity. Depth perception in these two ranges of disparity is referred to as “patent” and “qualitative” stereopsis (Ogle, 1952), respectively. All other things being equal, greater stereoscopic depth might be expected to enhance the 3D experience, by increasing the difference between the stereoscopic and non-stereoscopic experience.

A second factor that is enhanced under stereoscopic viewing, and which might also be expected to create a more convincing 3D experience, is the precision with which depth is represented. This precision also depends on the size of the disparity present in the stimulus. Ogle (1952) found that the standard deviation of errors, for a task in which the depth of a briefly presented target was aligned with that of a reference, increased exponentially with the disparity of the two stimuli relative to fixation. Blakemore (1970) measured relative disparity discrimination thresholds for distinguishing between the depth of a target and reference, for different values of reference disparity. He found that these thresholds increased exponentially up to 90 arc min, the largest value tested. Similar decreases in relative depth sensitivity with pedestal disparity have been reported elsewhere (Badcock & Schor, 1985).

Large separations intervals will create disparities beyond the range of qualitative stereopsis (Ogle, 1952). In this range, observers are not able to make even simple judgments as to which of two targets is closer than another, if fixation is kept fixed. If, however, observers are free to move their eyes, then accurate relative depth judgments can be made, relying partly on information about the change in convergence (Backus & Matza-Brown, 2003; Brenner and van Damme, 1998). The precision of relative depth judgments based on changes in convergence is not greatly affected by the magnitude of the disparity difference between the target objects (Brenner & van Damme, 1998).

It has previously been proposed that the realism of the 3D experience might relate to the precision with which depth is represented (Hibbard, 2008; Vishwanath, 2005). More specifically, it has also been proposed that quality of perceived depth is associated with the precision with which scaled metric depth is represented (Vishwanath, 2011, 2014).

A final factor that might be expected to affect the realism of the 3D experience is the extent to which the two images can be fused into a single percept. When the disparity in a stimulus is large, we are unable to completely fuse the two images together and as a result see some elements as double, an experience known as diplopia (Hampton & Kertesz, 1983; Qin, Takamatsu, & Nakashima, 2006). The range of single vision beyond which diplopia occurs is smaller than the range of patent stereopsis identified by Ogle (1952). This means that there is a range of disparities which are outside of Panum’s fusional limit and for which stimuli appear diplopic, but for which perceived depth nevertheless scales with disparity. There is also no direct link between the point at which fusion is lost and the precision of depth judgments. Rather, precision decreases at the same rate with increasing disparity both within and beyond Panum’s fusional limit (Wilcox & Allison, 2009). In complex stimuli, the limit of single vision is affected by many factors, including the size of the image (Hampton & Kertesz, 1983; Qin, Takamatsu, & Nakashima, 2006), how many elements it contains (Braddick, 1979; Burt & Julesz, 1980; Tyler, 1973), how long it is presented (Woo, 1974), and whether the observer is free to move their eyes (Mitchell, 1966). While a reduction in fusion might be expected to reduce realism for large disparities, it is unlikely to be able to account for variations in realism across the full range of disparities, particularly when the depth range is small. In the extreme, stimuli with zero or small disparities will have the greatest degree of fusion, but a low rating for creating a realistic depth experience.

Our goal was to test how the realism of the 3D experience from a stereoscopic display is associated with the magnitude and precision of perceived depth and with binocular fusion. Establishing these links is important in understanding the representational correlates of perceptual experience (Allen, 2013; Hibbard, 2008; Peacocke, 1987; Tye, 2002; Vishwanath & Hibbard, 2013; Vishwanath, 2014) and providing an optimal perceptual experience in stereoscopic applications (Häkkinen et al., 2008; IJsselsteijn et al., 1998; IJsselsteijn, de Ridder, & Vliegen, 2000; Lambooij et al., 2011; Seuntiens et al., 2006). To do this, we varied the disparity presented in stereoscopic images and measured how this affects the magnitude and precision of perceived depth, depth realism, and binocular fusion. To isolate the influence of binocular cues and reduce the effects of conflicts with pictorial cues, we used simple random element stereograms as our stimuli. We had a number of predictions about how these manipulations might affect the realism of depth.

We expected that there would be a range of disparities in which patent stereopsis would be apparent, such that apparent depth would increase with disparity. Beyond this range there should be no further increase in perceived depth. If 3D realism is determined by the magnitude of perceived depth, it should increase with disparity, within this range of patent stereopsis.

The sensitivity of observers to differences in depth was expected to decrease with increasing disparity. If 3D realism is determined by the precision of perceived depth, it should decrease with increasing disparity.

We also expected that, for disparities within Panum’s fusional limit, stimuli would be fused into a coherent percept, but for diplopia to occur for larger disparities. Binocular fusion might be expected to be important in establishing a convincing 3D experience with a sense of solid, 3D objects. We would then expect a convincing 3D experience when stimuli are fused and for this to diminish for large disparities, for which fusion does not occur. Equally, when disparities are small, and the stimuli do not differ substantially from those experienced with a non-stereoscopic display, we would not expect observers to report a strong 3D experience.

These predicted effects of binocular fusion and precision on depth realism are in the opposite direction from the predicted effects of depth magnitude. The predictions from fusion and depth sensitivity, while in the same direction, can also be distinguished. The prediction from fusion is that there will be a range of disparities over which fusion is maintained and for which a convincing 3D experience will be produced. Beyond this range, both fusion and realism will be lost. In contrast, if realism depends on the precision with which depth is represented, we expect depth sensitivity to decrease continuously both within and beyond the fusional limit, so would predict a continuous variation in the degree of realism. In particular, we would expect variation in the degree of realism even with small disparities, where there is no variation in the degree to which fusion is reported.

These experiments, by assessing the influence of disparity parameters on the realism of perceived depth, are related to previous studies (IJsselsteijn et al., 1998; Lambooij et al., 2011; Seuntiens et al., 2006). However, unlike these previous studies, which have used natural photographs, we used random circle stereograms to minimize the contribution of pictorial depth cues. This allowed us to separate the effects of disparity manipulations change the effects of conflicts between pictorial and binocular depth cues. Our stimulus manipulations are also linked to classic work which has established the influence of the range of disparities on the way that binocular images are fused together and depth is represented (Burt & Julesz, 1980; Ogle, 1952). In contrast to these studies, we focused on the effects of these manipulations on the subjective experience of the realism of 3D perception.

## Experiment one

In the first experiment, we varied the range of binocular disparities in stereoscopic stimuli to assess how this affected the apparent magnitude and experience of perceived depth. We chose a large range of disparities, up to 2.5 degrees, as we were interested in mapping the relationship between disparity and depth over a broad range. This range is beyond that typically considered as a comfortable depth budget in stereoscopic displays (Tam, Speranza, Yano, Shimono, & Ono, 2011). However, it was chosen on the basis of previous findings (Haines et al., 2015) as a range over which we expected participants to be able to make meaningful depth comparisons.

## Methods

### Apparatus

Stimuli were presented on a VIEWPIXX 3D monitor, viewed from a distance of 96 cm. The monitor screen was 52 cm wide and 29 cm tall. The screen resolution was 1920 × 1080 pixels, with a refresh rate of 120 Hz. Each pixel subtended 1 arc min. Stimuli were presented at 8-bit resolution. Stereoscopic presentation was achieved using a 3DPixx IR emitter and NVIDIA 3D Vision LCD shutter glasses. The cross-talk between the left and right images, measured using a Minolta LS-110 photometer, was 0.12%. Participants’ responses were recorded using a RESPONSEPixx response box. Stimuli were generated and presented using MATLAB and the Psychophysics Toolbox extensions (Brainard, 1997; Kleiner, Brainard, & Pelli, 2007; Pelli, 1997).

### Stimuli

**Fig. 1:**
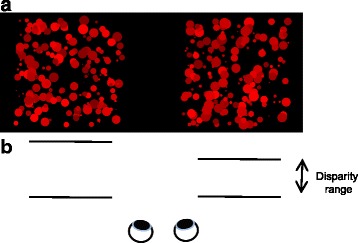
Example of the stimuli used. **a** The random-circle stereogram stimuli used. **b** For each of the stereograms the *dots* were presented on two planes, separated in depth. The observer’s task was to choose whether the stimulus on the *left* or the *right* contained the greatest depth separation or the greatest sense of depth realism. See text for the details of the stimuli used in each experiment

Each stimulus consisted of two stereograms, each containing a number of red circles (50 or 200 as detailed below), presented uniformly and randomly within a 10 × 10 degree square region (Fig. 1). The background of the display was black. The diameter of each circle was set at a randomly chosen value between 5 and 60 arc min, and its luminance at a randomly chosen value between 50% and 100% of the maximum possible value (100 cdm^–2^). In each stereogram, the circles were uniformly distributed across two depth planes. The circles on the front plane were presented with zero disparity and so appeared at the distance of the screen. The other plane was presented with uncrossed disparities and thus appeared behind the screen. The circles on the far plane of each stereogram were drawn before those on the near plane, so that occlusions were consistent with binocular depth cues. The distribution of disparities in our stimuli is thus much simpler than that typical of complex naturalistic scenes, but as a result allows for a simple manipulation of disparity and the effect this has on depth realism.

On each trial, two stereograms were presented. The stimuli were vertically centered on the screen, at eye-height, with one presented 7.7 degrees to the left of center and the other 7.7 degrees to the right.

For each stimulus, the uncrossed disparities of the back planes of the left and right stereograms were 15, 30, 60, 90, 120, or 150 arc min. In all cases, the disparities in the left and right stereograms were different. The number of circles in the stereograms varied across two blocks of trials and was either 50 or 200. For each number of dots, each of the 15 possible combinations of different disparities in the two stereograms was presented ten times within a block, and two blocks of trials were completed to give 20 repetitions of each pair of stimuli. Which of the two disparities was presented on the left and which on the right was randomized between trials.

### Procedure

On each trial, the stimulus was presented with an unlimited viewing time. Observers were free to move their eyes and change their convergence during this time. In separate sessions, the participant’s task was to report which of the two stereograms on each trial had the greater depth magnitude or greater sense of realism. When instructing the participants to judge realism, we asked them to consider in which of the two scenes the depth appeared the more tangible, solid, and real. Half the participants completed the depth magnitude trials first, the other half completed the realism trials first. All procedures were approved by the University of Essex Ethics Committee. Participants provided written informed consent to participate in the study.

### Participants

Ten participants (eight female, two male), including all three experimenters, completed the first experiment.

### Analysis

Participants’ responses were converted into scores on a Thurstone scale (Thurstone, 1927). For each of the 15 comparisons between two stereograms, each with a different disparity, the proportion of times that each disparity was chosen as having the greater depth magnitude in that pair, or greater 3D realism, was calculated. In performing these calculations, responses were combined across stimuli having the smaller disparity on the left and the right. Thus, for example, responses for stimuli with a disparity of 15 arc min on the left and 30 arc min on the right were combined with those for stimuli with a disparity of 30 arc min on the left and 15 arc min on the right. For two stimuli with disparities A and B, the number of times that each was chosen over the other, C_A,B_ and C_B,A_ was recorded. These counts were then used to calculate a measure of the difference in depth magnitude or realism between the two disparities:

1 $$ {D}_{A, B}={\phi}^{-1}\left(\frac{C_{A, B}}{C_{A, B}+{C}_{B, A}}\right) $$

where *ϕ* ^− 1^(*x*) is the inverse cumulative density function of the standard normal distribution.

For each disparity, its overall magnitude or realism score was then calculated as the mean score across its comparisons with all other stimuli:

2 $$ {S}_i=\frac{1}{N-1}{\displaystyle {\sum}_{\underset{i\ne j}{j-1}}^N{D}_{i, j}} $$

For counts of 0 and 20 values of 0.025 and 0.975 were used for the proportions in Eq. 1 to allow the calculation of the value of the inverse cumulative normal function (Tsukida & Gupta, 2011). Finally, since the resulting values depend on the number of repetitions, the scale values were normalized to the range of 0 (lowest possible value) to 1 (highest possible value), to allow the magnitude values to be directly interpreted.

## Results

**Fig. 2:**
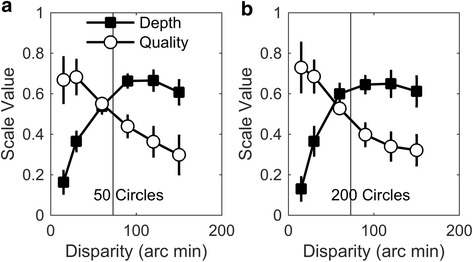
Mean depth magnitude and quality judgments for stimuli with (**a**) 50 and (**b**) 200 *circles*. The *vertical line* shows the mean point at which 50% of stimuli were seen as fused (as detailed in experiment 4). *Error bars* show ±1 standard error

Figure 2 plots the results for stereoscopic depth magnitude and realism judgments. These data were analyzed using mixed effects models, with disparity as a fixed covariate, dot number as a fixed factor, random intercepts, and random slopes for disparity. Apparent depth increased with increasing disparity (slope = 0.257; t(1,96) = 5.489; *p* < 0.001). There was no significant effect of dot number (t(1,96) = −0.04; *p* = 0.968) and no significant interaction (t(1,96) = 0.296; *p* = 0.768). The geometrically predicted increase in apparent depth with disparity tended to level off for large disparities, beyond around 60 arc min. Depth realism decreased with increasing disparity (slope = −0.0036; t(1,96) = 2.055; *p* = 0.043). There was no significant effect of dot number (t(1,96) = 0.288; *p* = 0.774) and no significant interaction (t(1,96) = 1.766; *p* = 0.081).

## Discussion

Perceived depth magnitude increased with disparity up to around 60 arc min, as expected. In contrast, depth realism decreased with increasing disparity. These results are clearly not consistent with the view that the degree of 3D realism is associated with an increase in the magnitude of depth perceived (Ames, 1925; Koenderink, van Doorn, & Kappers, 1994). They are however consistent with the idea that it is associated with the precision of depth perception (Hibbard, 2008), since we would expect that sensitivity to depth differences would decrease with increase disparity. In the second experiment, we tested this prediction directly.

## Experiment two

It is already well established that disparity discrimination thresholds increase with increasing disparity (Badcock & Schor, 1985; Blakemore, 1970; Ogle, 1952). The purpose of the second experiment was to demonstrate this effect for the particular stimuli and viewing conditions used here. This is particularly important as observers were free to change their convergence, so that disparity discrimination thresholds could not be predicted based on a pedestal disparity relative to a fixed convergence (Badcock & Schor, 1985; Blakemore, 1970; Smallman and Macleod, 1997).

## Methods

### Apparatus, stimuli, and procedure

The apparatus, general stimulus properties, and procedure were the same as in experiment one. Stereograms contained 100 elements, presented on two planes. One stereogram on each trial had a standard disparity of 15, 30, 60, 90, 120, or 150 arc min, corresponding to the disparities used in experiment one. The other stereogram had a test disparity that was the same as the standard, or was 5%, 10%, 15%, or 20% greater or smaller. The participant’s task was to decide which of the stimuli had the greater depth. In each block of trials, the standard disparity was fixed, and each test disparity was presented 40 times, in a randomized order.

### Participants

Six participants (five female, one male), including all three experimenters, completed the second experiment.

## Results

**Fig. 3:**
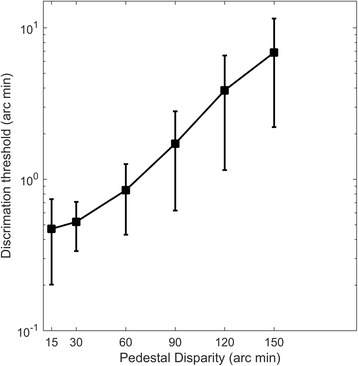
Mean 75% correct depth discrimination thresholds plotted as a function of the pedestal disparity. *Error bars* show ± standard error

A cumulative Gaussian curve was fit to participants’ responses, using the Palamedes toolbox (Prins & Kingdom, n.d.), with threshold (α) and slope (β) as free parameters. The depth discrimination threshold was defined as half the difference between the 25% and 75% points on this curve. These thresholds are plotted in Fig. 3. The data were analyzed using a mixed effects model, with the log of threshold as the dependent variable, the pedestal disparity as a fixed covariate, and random intercepts and slopes. Log threshold values were used given the exponential increase in threshold with increase pedestal that has been found (Ogle, 1952). Thresholds increased with increasing disparity (slope = 0.020; t(1,34) = 8.88; *p* < 0.001), as predicted.

## Discussion

Sensitivity to depth differences decreased with increasing disparity. This effect is consistent with previous results using simple stimuli, across a smaller range of disparities (Badcock & Schor, 1985; Blakemore, 1970; Ogle, 1952). We show that this decrease in sensitivity with increasing disparity holds for our stimuli, containing many stimulus elements, a broad range of disparities, and unrestricted viewing time and fixation. This increase was relatively modest for small disparity pedestals, and increased for larger values. Since our observers were free to change their fixation, this is consistent with earlier reports of a relatively modest effect of the pedestal disparity under these conditions (Brenner & Van Damme, 1998). For larger disparities, it is possible that disparity thresholds were also affected by conflicts between vergence and accommodation.

The large disparities in our stimuli, up to a maximum value of 2.5 degrees, are beyond the “depth budget” or “depth bracket” advised in stereoscopic applications. This value indicates the range of disparities that can be comfortably presented to the viewer (Shibata, Kim, Hoffman, & Banks, 2011; Tam et al., 2011; Winkler, 2014). Tam et al. (2011) calculated the largest range for this depth budget as ±1 degree. If we allow for the possibility that observers could fixate in the center of the disparity range, our disparities are just beyond the edge of this zone of comfort. Fixating at a different distance would create larger disparities, beyond the zone of comfort.

A consequence of using these large disparities is that they will have created diplopia, so that some of the elements will not have been fused into a single image and will have been perceived as double. It is difficult to put a clear value on the fusional limit for our stimuli from theoretical considerations, since this will have been affected by the facts that they were large (Hampton & Kertesz, 1983; Qin et al., 2006), contained many elements (Braddick, 1979; Burt & Julesz, 1980; Tyler, 1973), and were presented for an unlimited time (Woo, 1974) while participants were free to move their eyes (Mitchell, 1966).

## Experiment three

The presence of diplopia could potentially have affected participants’ judgments of depth magnitude (Ogle, 1952) and realism. We therefore repeated the experiments with a smaller range of disparities. In addition, we directly measured how fusion was affected by the magnitude of disparity for our stimuli.

## Methods

### Apparatus, stimuli, and procedure

The apparatus, general stimulus properties and procedure were the same as in experiments one and two. The maximum disparity in each stimulus was 5, 10, 15, 20, 25, or 30 arc min. The number of elements was fixed at 100 and they were presented on two planes. For the depth magnitude and 3D realism conditions, within a block of trials each of the 15 possible combinations of different disparity ranges was presented ten times. Two blocks of trials were completed for both the depth magnitude and realism conditions, to give 20 repetitions of each pair of stimuli in each case. The methods of experiment two were used to measure depth sensitivity for each of these stimuli.

### Participants

Ten participants, including experimenters PH and RH, completed the depth and realism judgments. Nine participants completed the depth sensitivity measurements.

## Results and discussion

**Fig. 4:**
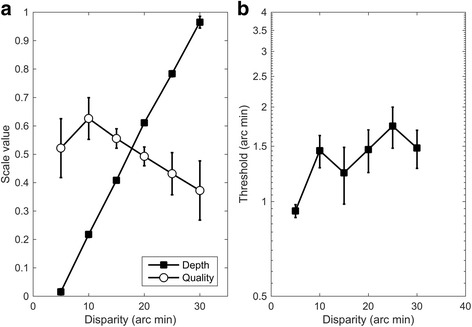
**a** Depth magnitude and quality values and (**b**) depth discrimination thresholds for experiment 3, in which a smaller range of disparities was used. Data plot the mean over ten participants; *error bars* show ±1 standard error

Figure 4a plots the perceived depth magnitude and realism against the smaller disparity range. Perceived depth increased significantly with increasing disparity (slope = 0.038; t(1,58) = 35.03; *p* < 0.001). There was no significant overall effect of disparity on depth realism (slope = −080; t(1,58) = −0.98; *p* = 0.331). In Fig. 4a, it can be seen that realism tended to increase from 5 to 10 arc min and then, similar to the large depth range, declined with disparity for larger values. However, even when this first point is excluded, this relationship is not significant (t(1,48) = −0.147; *p* = 0.147), so there is no evidence to support a relationship between disparity and realism with the small disparity range. Thresholds increased significantly with disparity (slope = 0.015; t(1,52) = 2.91; *p* = 0.0052).

For small variations in depth, realism did not therefore decrease as the precision of relative depth judgments decreased. The relationship between 3D realism and the magnitude and precision of perceived depth therefore differed between the large and small ranges of disparities used, possibly reflecting the difference in the extent to which the stimuli would have appeared fused.

## Experiment four

In the fourth experiment, we directly assessed the fusional range for our stimuli. This allowed us to determine whether the reduction in stereopsis was associated with a loss of fusion.

## Methods

### Apparatus, stimuli, and procedure

The apparatus and general stimulus properties were the same as in the previous experiments. The disparity separation in each stimulus varied between 5 and 150 arc min. The number of elements was fixed at 100 and they were presented on two planes. On each trial, a single stereogram was presented in the center of the screen and the participant was asked to judge whether it appeared fused. This was defined as the two depth planes appearing clear and distinct, with the individual elements solid and single. An unfused stimulus was defined as one in which the depth was less distinct and, in particular, one in which the elements may appear doubled or lustrous. Example stimuli, with disparity separations of 7 and 120 arc min, were presented before the experiment to ensure that the participants understood these criteria. Within a block of trials, each stimulus was presented ten times. Four blocks of trials were completed, so that each stimulus was presented 40 times.

### Participants

Eleven participants (five female, six male), including experimenter PH, completed the fourth experiment.

## Results

**Fig. 5:**
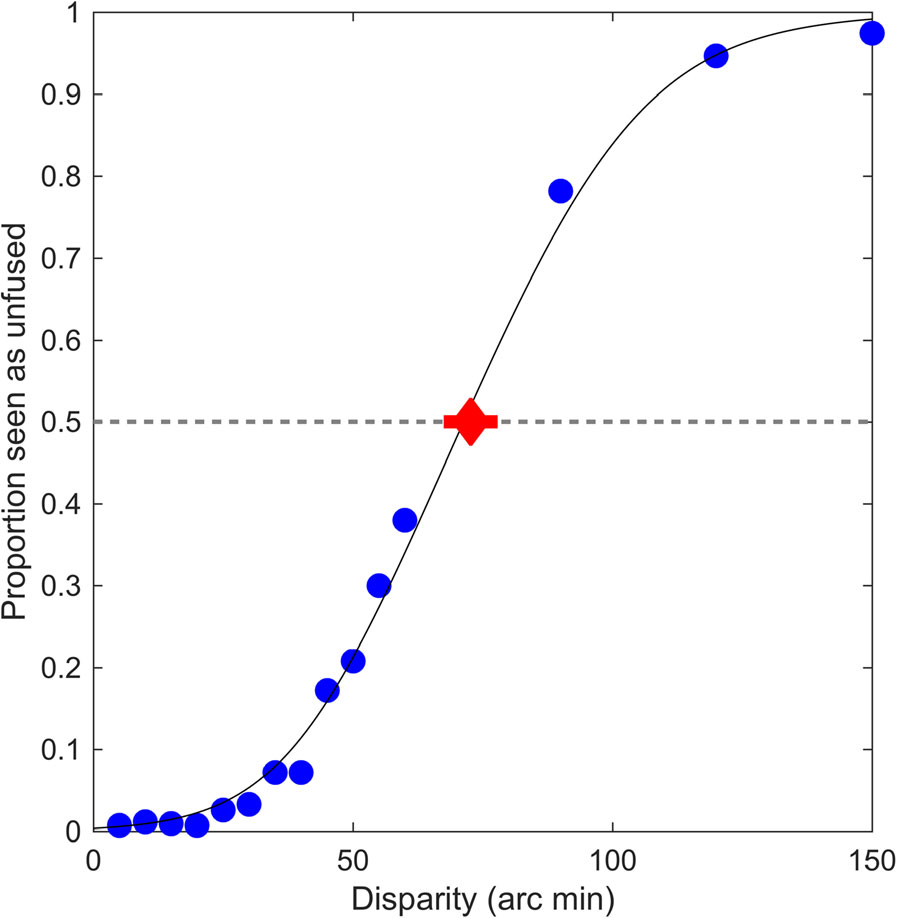
Proportion of stimuli seen as unfused, as a function of the disparity separation between the two planes. The *blue dots* show the mean proportion of unfused responses across participants, the *black curve* the average of the individual psychometric curve fits. The *red diamond* shows the mean 50% points, across participants; the *horizontal error bar* indicates ±1 standard error

The mean proportion of trials that were perceived as fused is plotted in Fig. 5 as a function of disparity. As expected, stimuli containing a small disparity were almost always perceived as fused, while those containing a large disparity were almost always perceived as unfused. We fit a cumulative Gaussian curve to the data, separately for each observer, to find the disparity at which 50% of stimuli were perceive as fused. The mean value, 73 arc min, is also plotted in Fig. 5.

## Discussion

The mean disparity value, across participants, at which the stimulus was judged as fused on half of trials, was 73 arc min. This value is plotted as the vertical line in Fig. 2. This corresponds to the range of disparities for which apparent depth no longer increased with disparity for our stimuli, consistent with the idea of patent stereopsis occurring within the range of diplopia. The decrease in fusion across the range of disparities is also reflected in the decrease in realism for disparities beyond 30 arc min, consistent with the view that diplopia might contribute to a reduction. However, the precision of relative depth judgments decreased in the same way, meaning that any attempt to disentangle the contributions of these two factors to stereopsis is not straightforward. In the following section, we summarize the results of all four experiments and how each of the factors that we have manipulated might contribute to the overall quality of the depth experience.

## Overall summary

We considered how depth realism was related to the magnitude of depth perceived, the precision of its representation, and the experience of diplopia. In this section, we consider the contributions of this range of factors across all four of our experiments.

### Geometrically predicted depth magnitude

The magnitude of depth that should be perceived in each stimulus can be predicted from the convergence distance to the screen, which is the distance at which the front plane of dots is presented, and the uncrossed disparity of the dots on the back plane. It has been suggested that depth realism should increase with apparent depth (Ames, 1925; Koenderink et al., 1994). For each disparity, the geometrically predicted depth was calculated assuming an interocular distance of 63 mm.

### Perceived depth magnitude

Since perceived depth does not correspond directly with that predicted geometrically, we also used the scale of relative apparent depth derived through pairwise comparisons as an alternative measure of depth magnitude.

### Precision of depth discrimination

Depth discrimination thresholds were used as a measure of the precision of relative depth judgments. Depth realism might be expected to increase as the precision of relative depth judgments increases.

### Binocular fusion

We used the mean number of times that each stimulus was chosen as unfused as a measure of binocular fusion. Stereopsis is expected to decrease as the degree of fusion decreases.

For each of these predictors a linear regression was performed, separately for each depth range, and the coefficient of determination, r^2^, was calculated across the realism values, and residuals, for the two disparity ranges combined (Table 1).

**Table 1:** Coefficients of determination for predicting depth realism

Predictor	r^2^
Geometrically predicted depth	0.738
Apparent depth	0.704
Depth discrimination threshold	0.7484
Fusion	0.9933

$$ {r}^2=1-\frac{{\displaystyle {\sum}_{i=1}^m}{\left({}_i^l R-{}_i^l P\right)}^2+{\displaystyle {\sum}_{i=1}^n}{\left({}_i^s R-{}_i^s P\right)}^2}{{\displaystyle {\sum}_{i=1}^m}{\left({}_i^l R-\overline{{}^l R}\right)}^2+{\displaystyle {\sum}_{i=1}^n}{\left({}_i^s R-\overline{{}^s R}\right)}^2} $$

where *R* is the realism value based on observers’ responses (and $\overline{R}$ is the mean across disparities), *P* is the value predicted from the linear regression, and the superscripts *l* and *s* refer to the large and small depth ranges, respectively. A single regression could not be performed for each predictor, across the two depth ranges, since the realism values for each stimulus can be interpreted only relative to the other stimuli to which it was compared. The coefficient of determination here quantifies the proportion of variance accounted for by the regression analyses across the two disparity ranges combined. Geometrically predicted or apparent depth, and discrimination thresholds, all gave similarly good predictions and the best prediction was given by the degree of fusion. Together, these results show that realism tended to increase as (1) depth decreased, (2) discrimination thresholds decreased, and (3) fusion increased. It is not possible to uniquely associate realism with any one of these predictors, since they are strongly correlated. As disparity increases, perceived depth increases and depth sensitivity and fusion decrease. However, while realism and thresholds change at a constant rate between 10 and 150 arc min, there is an abrupt loss of fusion between 40 and 100 arc min. These results thus suggest that precision is more closely related to realism that is fusion.

## General discussion

We assessed how depth realism was associated with the amount of depth perceived, the precision with which it is represented, and the degree to which stimuli could be fused into a coherent percept. Depth realism tended to decrease with increasing disparity. This is consistent with the decrease in both the precision of depth perception and binocular fusion with increasing disparity.

Our results are consistent with the view that the quality of perceived depth is related to the determinacy with which it is represented (Tye, 2002). This is relevant to the Transparency Thesis (Harman, 1990; Martin, 2002) which states that, when we reflect on our perceptual experience, we are only aware of “mind-independent” objects. In particular, this thesis argues that we are not aware of properties of the experience itself (Allen, 2013). A broader view, known as openness, (McDowell, 1994) is that perceptual experience can consist of both mind-independent, and non-mind-independent properties. As an example, Allen (2013) discusses the case of blurred visual experiences. When we have a blurred experience of seeing an object, the object itself may be thought of as mind-independent, but the blurriness is a property of our experience and therefore not mind-independent. Allen (2013) accounts for blur as a form of “over-representation;” rather than representing the location of each edge of an object as lying in single location, he argues that we represent it as being simultaneously at multiple locations. This thus creates uncertainty about the exact location of the edge – it is represented not as being located at a single, determinate position, but within a range of possible positions. The notion of over-representation is related to Bayesian models of perception (Knill & Richards, 1996). In a Bayesian model, perceptual estimates take the form of probability distributions, rather than single, determinate values. For example, based on the available visual evidence and our prior assumptions, we can calculate the posterior probability density function, describing the relative likelihood that the edge is located at each possible spatial position. Under this description, a sharp visual experience would correspond to a posterior density function that is tightly focused around the actual location of the edge, while a blurry experience would correspond to one in which the density function if more broadly distributed. In the case of depth perception, there is a distinction between the amount of depth that the object is seen to have (a mind-independent property) and the realism or convincingness of the depth experience (a mind-dependent property). Both of these aspects of the perceptual experience are important considerations in 3D displays, allowing for effective interaction with the scene and a strong sense of presence, respectively.

Lambooij et al. (2011) argued that a quality metric for 3D should capture aspects of both image quality and the depth experience. Here, we argue that the depth component in turn needs to be carefully separated into multiple dimensions, including magnitude and realism. Techniques for the measurement of the magnitude and veridicality of depth are well developed (Koenderink, 1998) and have been successfully applied to stereoscopic displays (Doorschot, Kappers, & Koenderink, 2001; Hornsey, Hibbard, & Scarfe, 2015). We have shown here that participants are clearly able to distinguish between magnitude and realism judgments of depth when making more subjective assessments.

Our stimuli consisted of arrays of randomly positioned circles, presented on two planes in depth defined by binocular disparity and occlusion. These stimuli, rather than complex naturalistic scenes, were used to provide simple disparity content which could be easily quantified and to minimize the contribution of pictorial depth cues. It should be noted, however, that some conflict between binocular and monocular cues for a typical stereoscopic display such as ours is inevitable. For example, both focus cues and the range of sizes of circles on the two planes were both consistent with there being no depth separation between the two planes.

Our results show that, for our stimuli, depth realism improved with increasing disparity over only a very small range, of less than 15 arc min. This is much less than the typical depth budget of around 1 degree, recommended in stereoscopic applications, which tend to be motivated by considerations of viewing comfort and fatigue (Kuze & Ukai, 2008; Lambooij, IJsselsteijn, & Heynderickx, 2007; Shibata et al., 2011; Speranza, Tam, Renaud, & Hur, 2006; Tam et al., 2011; Yano, Ide, Mitsuhashi, & Thwaites, 2002). From considerations of depth realism, we would recommend an approach of “just enough reality” (Siegel & Nagata, 2000) and using only enough disparity to optimize the quality of the experience. Larger values of disparity, while leading to the perception of a greater amount of depth, tend to reduce the realism with which it is perceived and increase the likelihood of discomfort.

## Conclusions

A realistic and convincing sense of depth is important in creating a rewarding experience and a sense of presence and immersion in stereoscopic displays and virtual reality. We assessed how realism of depth in displays related to the amount of depth perceived, the precision of depth judgments, and the fusion of the display into a coherent percept. We used simple stereoscopic stimuli, containing two planes in depth, to determine how each of these attributes was affected by the amount of binocular disparity in the stimuli. Depth magnitude tended to increase with disparity, while realism, precision, and fusion tended to decrease. In the field of stereoscopic displays, if the goal is to produce a realistic depth experience, our results suggest that the optimal strategy is to provide just enough disparity to create a convincing stereoscopic effect.
